# Supplementary material for: The limitations of phenotype prediction in metabolism
Source: PLoS Comput Biol. 2023 Nov 10;19(11):e1011631. doi: 10.1371/journal.pcbi.1011631 (PMC10664875; doi:10.1371/journal.pcbi.1011631)
Supplement: S1 Text — Supplementary note including a brief introduction to flux balance analysis, global sensitivity analysis, a detailed discussion on the additivity in genotype to phenotype metabolic maps and the GO enrichment results of predictors with the largest effect sizes. (PDF) [file pcbi.1011631.s001.pdf]

Supporting Text

The limitations of phenotype prediction in metabolism

Pablo Yubero, Alvar A. Lavin, Juan F. Poyatos.
Logic of Genomic Systems Lab, CNB-CSIC, Madrid, Spain.

Flux Balance Analysis

Flux balance analysis (FBA) is a computational technique that allows the simulation of metabolism from information about its structure. This information is now available for many organisms through genome-scale reconstructions of metabolic networks. Networks are represented in this formalism by a stoichiometric matrix of metabolites and reactions. Using computational methods, FBA calculates an optimal solution which comprises the quantitative values of the fluxes through all reactions. Such a solution is obtained by optimizing an objective function, which in most cases is the maximization of biomass production. Prior to this calculation, the flows of these reactions can be restricted, which ultimately delimit the space of possible solutions influencing the predicted phenotype. Also, nutrient exchange reactions are included in metabolic models and their bounds define the growth medium of the metabolism.

While acknowledging that genetic regulation is an important aspect and a known limitation of metabolic models, in our analysis, we make the assumption that regulatory mechanisms are functioning appropriately and unhindered. We consider the possibility that all possible and known alternate biosynthetic routes can be eventually required and activated to support growth. By adopting this approach, we aim to capture the potential metabolic capabilities of the system under different genetic variations. Although our study does not explicitly incorporate the genetic regulation aspect, we firmly believe that our approach still yields valuable insights into the predictability of growth based on genetic variation. Please refer to [1] for a primer description of this formalism.

Global sensitivity analysis

Global sensitivity analysis (GSA) is a mathematical toolset that contributes to the interpretability of complex models by decomposing an output variance into partial variances of input variables, or subsets of these. These methods have rapidly grown and have been widely used, for example in risk and model assessment. They have also been previously used in the context of flux balance analysis, but to assess the contribution of reactions instead of genes [2]. We summarize in the following the main ideas behind Sobol's approach to GSA and the protocol that we used [3–6]. Imagine any model that takes p input parameters $\vec{x} = \{x_i\}_{i=1}^{p}$ and outputs a scalar value $y = f(\vec{x})$. Here parameters and variables can be considered equivalent. Then the variance of the output $\mathrm{Var}(y)$ can be decomposed as:

$$\mathrm{Var}(y) = \sum_{i=1}^{p} V_i + \sum_{i=1}^{p} \sum_{j>i}^{p} V_{ij} + \ldots + V_{\vec{x}}, \tag{1}$$

which, dividing all terms by $\mathrm{Var}(y)$, can be simplified and rewritten as a function of the Sobol *indices* S:

$$1 = \sum_{i=1}^{p} S_0^i + \sum_{i=1}^{p} \sum_{j>i}^{p} S_{ij} + \ldots + S_{\vec{x}}. \tag{2}$$

This decomposition is particularly revealing as S_0 are the first order, fractional, contributions of each individual

parameter to the total variance, while the rest include contributions of gradually increasing order, i.e. interactions between pairs of parameters, then triplets, etc. Apart from S_0, the total effects index is of particular interest, and it can be written as:

$$S_T^i = \sum_{\vec{w}_i} S_{\vec{w}_i}, \tag{3}$$

where all $\vec{w}_i$ contain the i-eth parameter. In this way, S_T^i quantifies the fraction of variation associated to the i-eth parameter and all of its interactions with other parameters.

To compute these values, common approaches include Monte Carlo estimates and Fourier amplitude estimate testing [5]. We focus on the former due to its simplicity and satisfactory convergence. Among different Monte Carlo estimators [6], we used the following for S_0 and S_T:

$$S_0^i = \frac{1}{N\, \mathrm{V}ar\,(f(A))} \sum_{k=1}^{N} f(B^k)\left(f(A_{Bi}^k) - f(A^k)\right), \text{ and} \tag{4}$$

$$S_T^i = \frac{1}{2N\, \mathrm{V}ar\,(f(A))} \sum_{k=1}^{N} \left(f(A^k) - f(A_{Bi}^k)\right)^2, \text{ with} \tag{5}$$

$$\mathrm{Var}\,(f(A)) = \frac{1}{N} \sum_{k=1}^{N} f(A^k)^2 - \left(\frac{1}{N} \sum_{k=1}^{N} f(A^k)\right)^2, \tag{6}$$

where k is the sample, N is the total number of samples, $f(A^k)$ is the growth rate of genotype A^k, $f(B^k)$ is that of genotype B^k, and $f(A_{Bi}^k)$ is that of genotype A^k but with the dosage of the i-eth gene taken from genotype B^k. Also, A and B are genotypes sampled from our default population.

Therefore, the Monte Carlo protocol can be summarized in the following steps per sample:

1. Obtain two genotypes $A = \{g_i^A\}_{i=1}^l$ and $B = \{g_i^B\}_{i=1}^l$, where l is the number of genes.

2. Create l new genotypes $\{A_B^i\}_{i=1}^l$ such that all dosages are from A except for the i-eth which is taken from B, that is $A_B^i = (g_0^A, g_1^A, ..., g_{i-1}^A, g_i^B, g_{i+1}^A, ..., g_l^A)$.

3. Compute $f(A)$, $f(B)$ and $f(A_B^i)$ for i=0,...,l.

4. Use Eq.(4) and Eq.(5) to compute S_0 and S_T, respectively.

Additivity on genotype to phenotype metabolic maps

Our goal here is to clarify the apparent contradiction between the substantial fraction of additivity found in the genotype-phenotype (GP) metabolic map and the relatively small R2 values of the associated PGS. According to Gjuvsland et al. [7,8], the dosage-response monotonicity, or order preservation, of a GP map leads to a significant

fraction of the additive variance that should favor predictability (R^2 large). To begin with, we note that the monotonicity of the metabolic reconstruction is highly additive. Order conservation is not "broken" in any case (dosage-response profiles in S7 Fig show degree of monotonicity m=1, following [8]). In addition, we show through the global sensitivity analysis that the sum of the first order indices represents about 75% of the total variance. Therefore, our model has a substantial fraction of additive variance.

Why, then, is R^2 =0.27? We argue that the R^2 values we found are the result of a trade-off between the order-preserving nature of the metabolic GP maps and the population substructure in terms of which genes are "predictors" in each individual [9]. First, the GP map is not fully additive despite showing full monotonicity since the dosage-response curves show a general pattern of partial dominance (this does not analytically contradict that m=1, but could reflect a possible limitation of such measure). Second, the prediction of the phenotype using a training population is certainly population dependent. In the main text, we show that in these models, predictors arise when they are rate-limiting, that is, when they effectively limit the flux through the biomass reaction (the phenotype). Which enzyme is rate-limiting depends on the individual and their genetic background (Fig. 7, main text). To illustrate this, we sought to identify which specific enzymes limit growth in each individual from a small population sample. We do this by calculating the growth costs produced by small "virtual" mutations in each individual and in each enzyme sequentially. Ideally, virtual mutations would be infinitesimal in likeliness with virtual displacements in Classical Mechanics.

In S8 Fig, we show the results of these growth costs (rows; enzymes that never appear as limiting –rows that have only 0s– not shown) in 10^3 different individuals (columns) in two populations, one in which the performance of the PGS is worse than the other (S8A Fig with R^2 = 0.26 and S8B Fig with R^2 = 0.84). By applying an agglomerative clustering algorithm, we identified the population substructure as early as 10^3 individuals: there is a broader structure in the dendrogram of S8 Fig panel B than in panel A. This explicitly demonstrates that the PGS not only depends on the additivity of the GP map itself, but also loses predictive power due to the integration of results from different subpopulations (this is reminiscent of similar discussions focusing on epistasis, e.g., [10]). The number of predictors in a population is, in our work, an indicator of the number of subpopulations present. Therefore, the smaller the number of subpopulations, the better the performance of the PGS (see also Fig 7C, main text).

GO enrichment analysis results

GO term	Cluster frequency	Genome frequency	p-value (corrected)	FDR	False positives
cellular BP	100,0%	55,6%	5,01E-07	0,0%	-
organic substance BP	100,0%	58,5%	2,76E-06	0,0%	-
BP	100,0%	59,2%	4,03E-06	0,0%	-
histidine BP	21,9%	1,2%	5,54E-07	0,0%	-
histidine MP	21,9%	1,2%	5,54E-07	0,0%	-
lipid BP	43,8%	10,0%	4,23E-05	0,0%	-
organic acid BP	59,4%	20,1%	8,53E-05	0,0%	-
carboxylic acid BP	59,4%	20,1%	8,53E-05	0,0%	-
small molecule BP	68,8%	30,0%	5,30E-04	0,0%	-
glycerolipid BP	21,9%	2,5%	5,70E-04	0,0%	-
glycerophospholipid BP	21,9%	2,5%	5,70E-04	0,0%	-
lipid MP	46,9%	14,1%	6,20E-04	0,0%	-
cellular lipid MP	46,9%	14,1%	6,20E-04	0,0%	-
glycerophospholipid MP	25,0%	3,6%	6,90E-04	0,0%	-
glycerolipid MP	25,0%	3,7%	9,40E-04	0,0%	-
phospholipid BP	25,0%	4,0%	1,68E-03	0,0%	-
alpha-amino acid BP	43,8%	13,7%	2,56E-03	0,0%	-
phospholipid MP	21,1%	5,9%	4,60E-03	0,0%	-
cellular amino acid BP	43,8%	14,8%	6,37E-03	0,0%	-
GDP-mannose BP	9,4%	0,4%	8,85E-03	0,1%	0,02
GDP-mannose MP	9,4%	0,4%	8,85E-03	0,1%	0,02
long-chain fatty acid BP	9,4%	0,4%	8,85E-03	0,1%	0,02

Table A. GO enrichment analysis of predictors with the largest effect sizes. We further confirm that the top genetic predictors cluster into only a few biosynthetic and metabolic processes (BP and MP, respectively). They are mainly related with amino acids, phospholipids, fatty acids and mannose [11].

References

1. Orth JD, Thiele I, Palsson BØ. What is flux balance analysis? Nature Biotechnology. 2010;28(3):245–248. doi:10.1038/nbt.1614.

2. Nobile MS, Coelho V, Pescini D, Damiani C. Accelerated global sensitivity analysis of genome-wide constraint-based metabolic models. BMC bioinformatics. 2021;22(Suppl 2):78–78. doi:10.1186/s12859-021-04002-0.

3. Sobol IM. Sensitivity analysis for non-linear mathematical models. Mathematical modelling and computational experiment. 1993;1:407–414.

4. Sobol IM. Global sensitivity indices for the investigation of nonlinear mathematical models. Matematicheskoe Modelirovanie. 2007;19(11):23–24.

5. Saltelli A, Ratto M, Andres T, Campolongo F, Cariboni J, Gatelli D, et al. Global Sensitivity Analysis: The Primer. Wiley; 2008.

6. Saltelli A, Annoni P, Azzini I, Campolongo F, Ratto M, Tarantola S. Variance based sensitivity analysis of model output. Design and estimator for the total sensitivity index. Computer Physics Communications. 2010;181(2):259–270. doi:10.1016/j.cpc.2009.09.018.

7. Gjuvsland AB, Vik JO, Woolliams JA, Omholt SW. Order-preserving principles underlying genotype–phenotype maps ensure high additive proportions of genetic variance. Journal of Evolutionary Biology. 2011;24(10):2269–2279. doi:https://doi.org/10.1111/j.1420-9101.2011.02358.x.

8. Gjuvsland AB, Wang Y, Plahte E, Omholt SW. Monotonicity is a key feature of genotype-phenotype maps. Frontiers in Genetics. 2013;4. doi:10.3389/fgene.2013.00216.

9. Hill WG, Goddard ME, Visscher PM. Data and Theory Point to Mainly Additive Genetic Variance for Complex Traits. PLOS Genetics. 2008;4(2):1–10. doi:10.1371/journal.pgen.1000008.

10. Mackay TFC. Epistasis and quantitative traits: using model organisms to study gene–gene interactions. Nature Reviews Genetics. 2014;15(1):22–33. doi:10.1038/nrg3627.

11. Cherry JM, Hong EL, Amundsen C, Balakrishnan R, Binkley G, Chan ET, et al. Saccharomyces Genome Database: the genomics resource of budding yeast. Nucleic Acids Res. 2012;40(Database issue):D700–5.
