# Supplementary figures and images for: The limitations of phenotype prediction in metabolism
Source: PLoS Comput Biol. 2023 Nov 10;19(11):e1011631. doi: 10.1371/journal.pcbi.1011631 (PMC10664875; doi:10.1371/journal.pcbi.1011631)

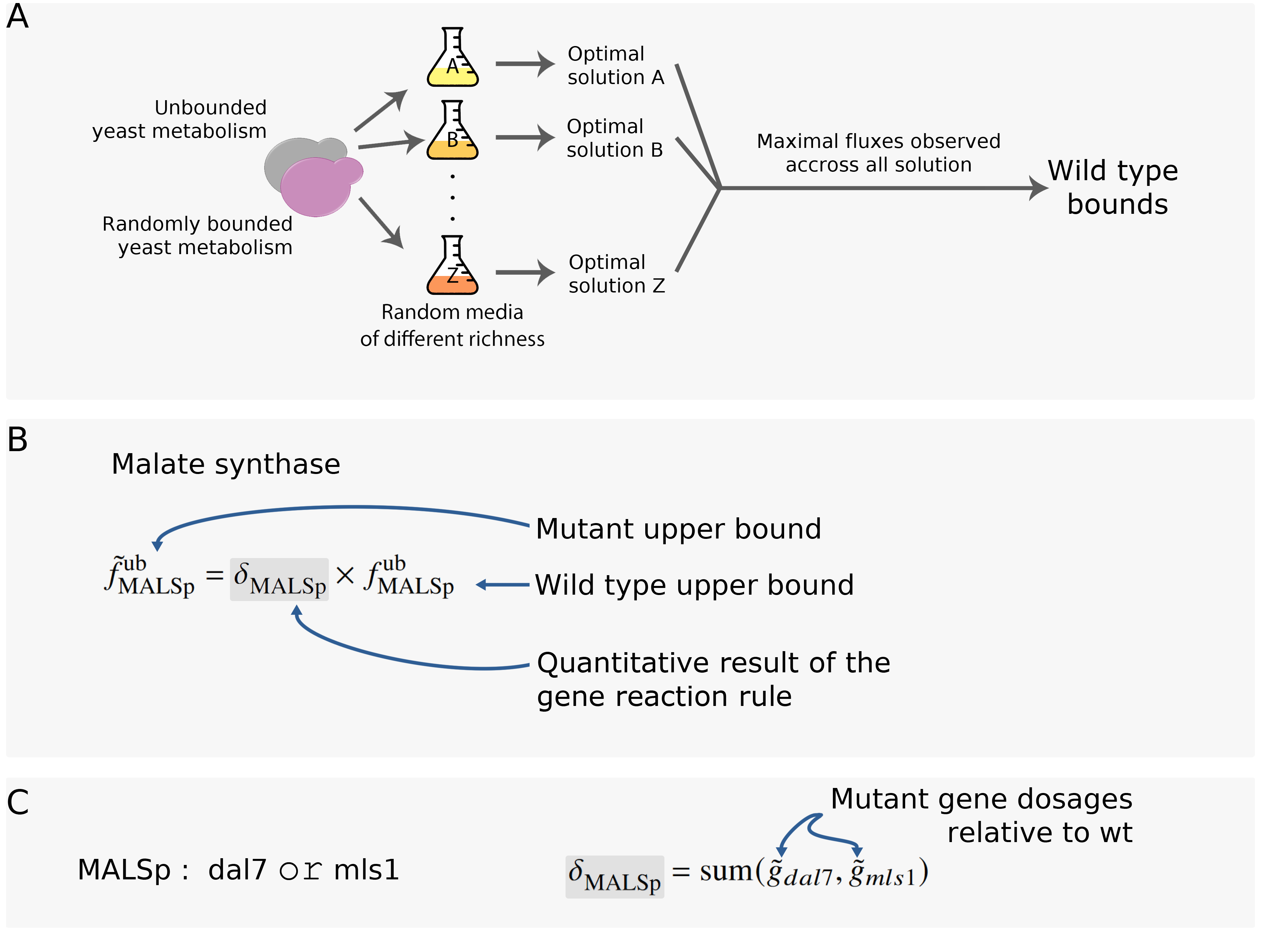

Supplement: S1 Fig — We characterize mutations by a decrease in enzyme efficiency with respect to a wild-type “reference”, or “maximum” value. A: To find the wild-type lower and upper bounds of each reaction r, frlb and frub, we expose the yeast metabolism to a series of environmental and genetic conditions and compute the minimum and maximum fluxes observed in the solutions taking into account their reversibility. Specifically, we compute pairs of optimal solutions in 104 random media from a totally unbounded and a randomly bounded yeast metabolisms (random bounds change in every medium; Methods). The bound for a given mutant and reaction is the product of the corresponding wild-type bound and a fractional value resulting from the quantitative interpretation of the associated gene reaction rule. B: Here we show a detailed example involving the malate synthase reaction MALSp which is mediated by two isozymes dal7 and mls1. The mutant upper bound, f˜MALSpub where ˜ denotes mutant, is the product of the wild-type upper bound fMALSpub and the result of the gene reaction rule δMALSp. C: The gene reaction rule in the case of MALSp reads “dal7 or mls1”, so we compute the sum of the relative gene dosages in the mutant of enzymes dal7 and mls1, g˜dal7 and g˜mls1 respectively. (TIF) [file pcbi.1011631.s002.tif]

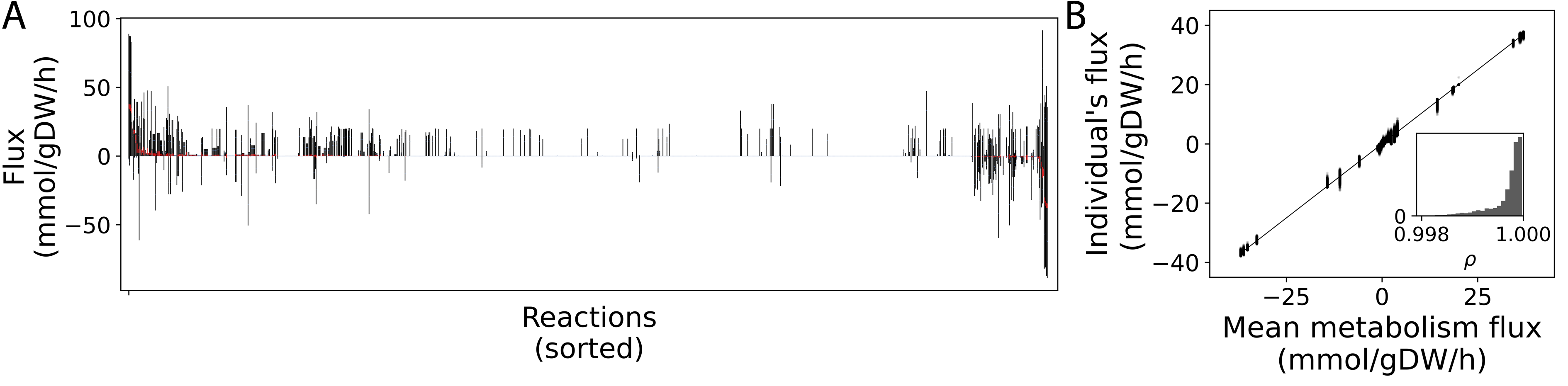

Supplement: S2 Fig — A: Fluxes that are accessible in the population, i.e. maximal bounds (vertical black lines) and range of values observed in the population (vertical red lines) for each reaction (x-axis). Blue lines represent 70% of such maximal bounds, which is approximately the largest restriction in the default population (with dosages sampled from a normal distribution with unit mean and σ = 0.1). We find that the genetic variation with which the population was generated leads to variability in some solution fluxes of the individuals, which ultimately translate into growth variability. B: Despite this variability in solution fluxes, we can define a “mean” metabolism in which the flux through each reaction is the observed mean across the population. Black dots depict data of the reactions of all individuals in the population, and the inset shows the distribution of linear correlations between each individual’s solution and the mean metabolism. (TIF) [file pcbi.1011631.s003.tif]

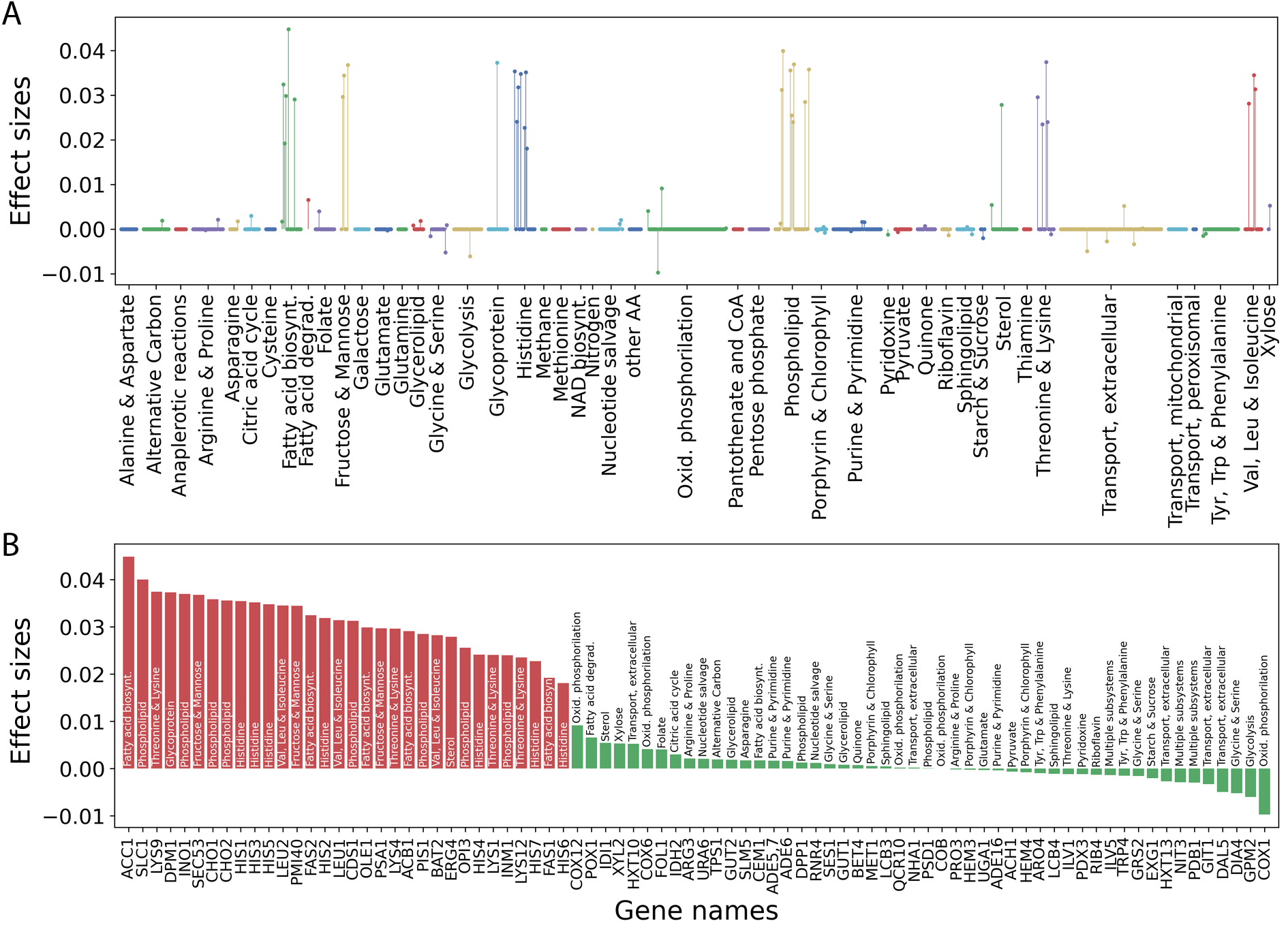

Supplement: S3 Fig — A: Manhattan-like plot showing the effect sizes (y-axis) of genes grouped by yeast metabolic subsystems (x-axis; arbitrary colors). We find that genes with large effect sizes belong to a handful of subsystems related to protein synthesis, cell membrane and organelle compartmentalization. B: Effect sizes of all predictors identified in the PGS (colors as in Fig 2) and their corresponding metabolic subsystem. (TIF) [file pcbi.1011631.s004.tif]

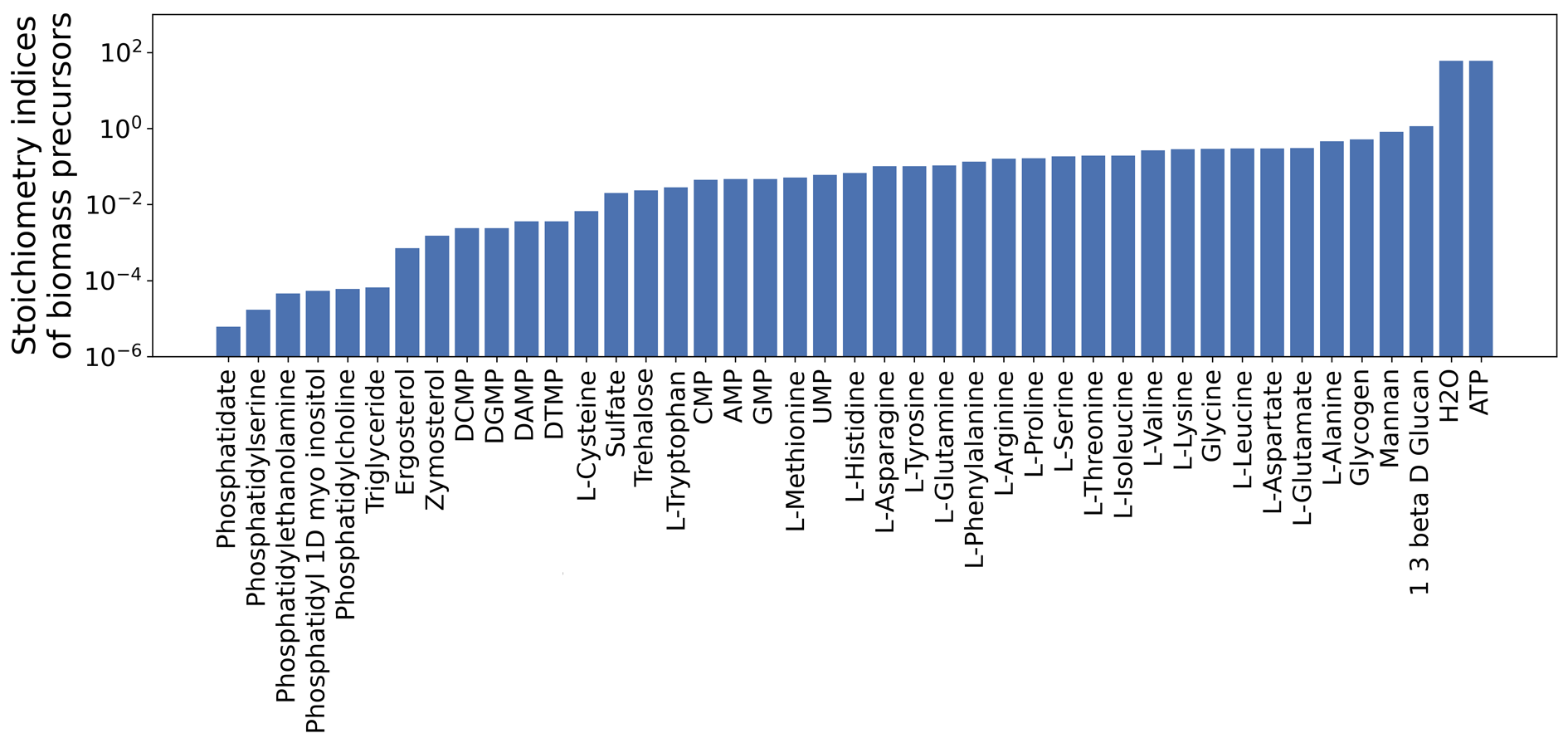

Supplement: S4 Fig — The biomass reaction involves 43 precursor metabolites (x-axis) but with stoichiometric coefficients spanning several orders of magnitude (y-axis, in log scale). For example, the most consumed precursors are ATP and water. (TIF) [file pcbi.1011631.s005.tif]

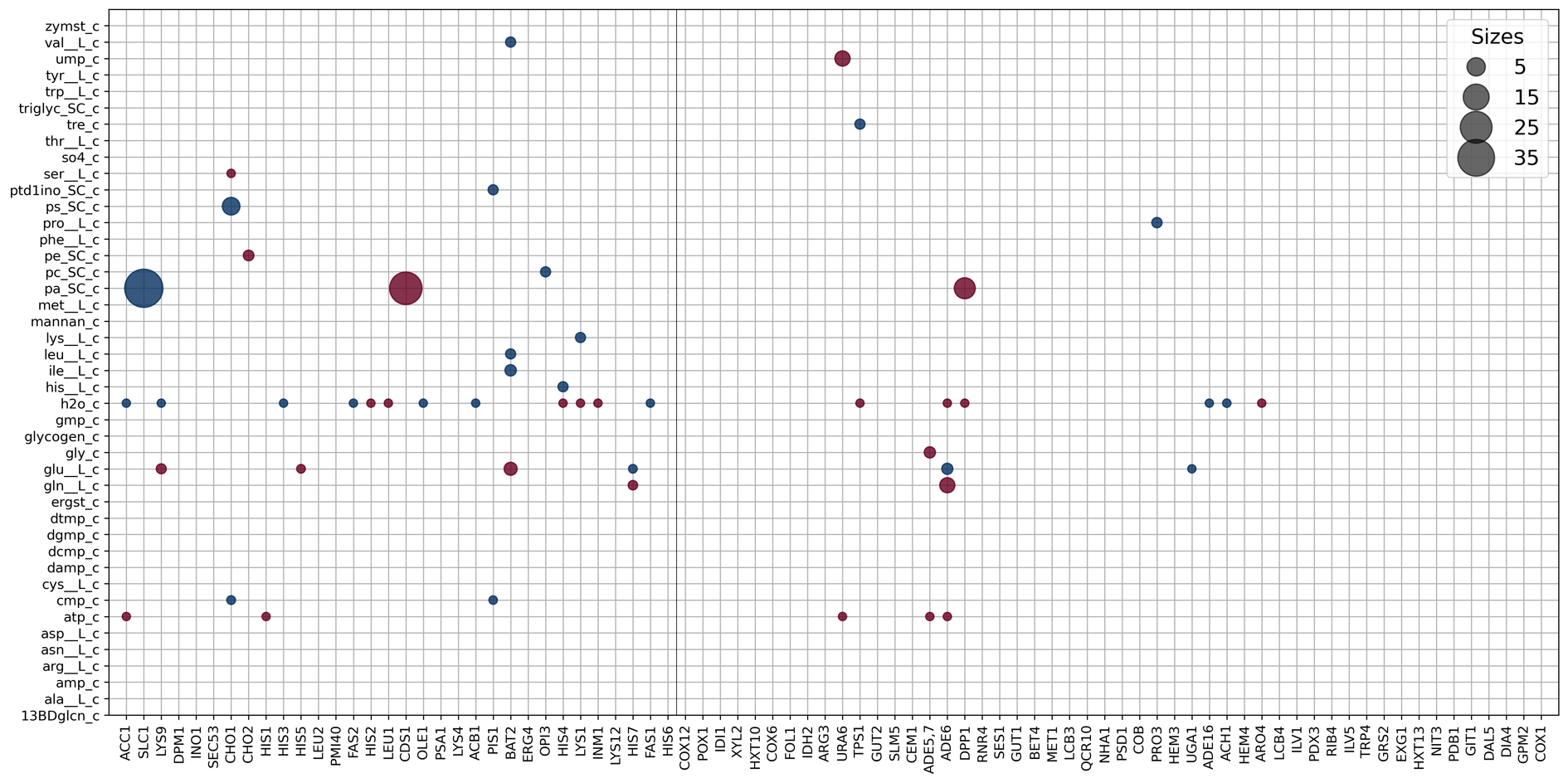

Supplement: S5 Fig — Each genetic predictor (x-axis; sorted by effect size) participates in a number of reactions that might involve biomass precursors (y-axis). We here show the mean consumption (red circles) or production (blue circles) across the entire population (104 individuals). Circle sizes are proportional to the absolute value of the mean contribution relative to the biomass consumption. (TIF) [file pcbi.1011631.s006.tif]

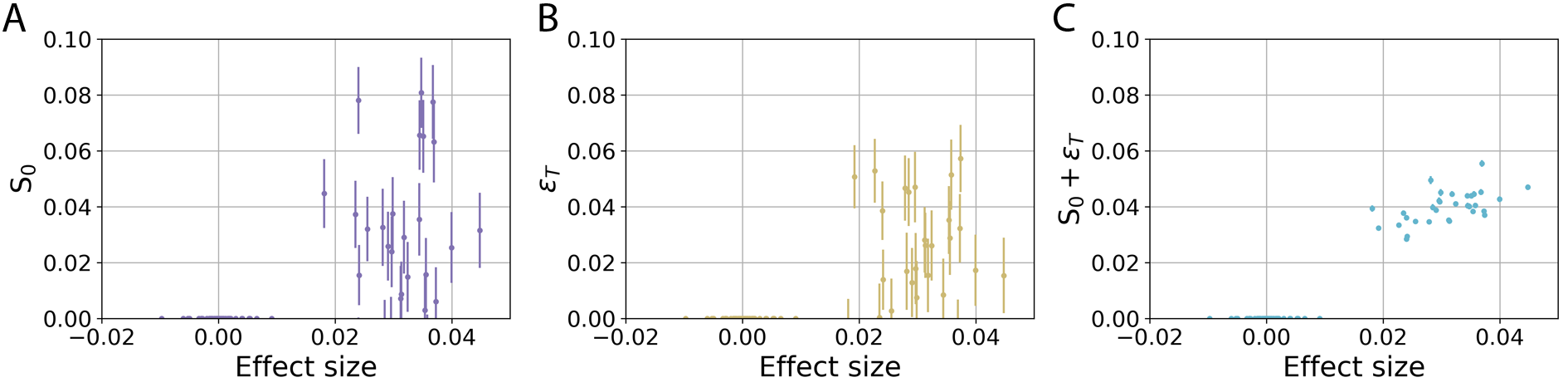

Supplement: S6 Fig — A: first order index S0, B: total epistasis ϵT and C: total effects S0 + ϵT as a function of effect size (Methods). The linear correlations among all genes are ρS0all=0.63, ρϵTall=0.48 and ρ+all=0.98 respectively; or among only large effect predictors ρS0pred=0.19, ρϵTpred=-0.08 and ρ+pred=0.57. We show the mean values and a standard deviation of > 106 simulations for each gene (Methods). (TIF) [file pcbi.1011631.s007.tif]

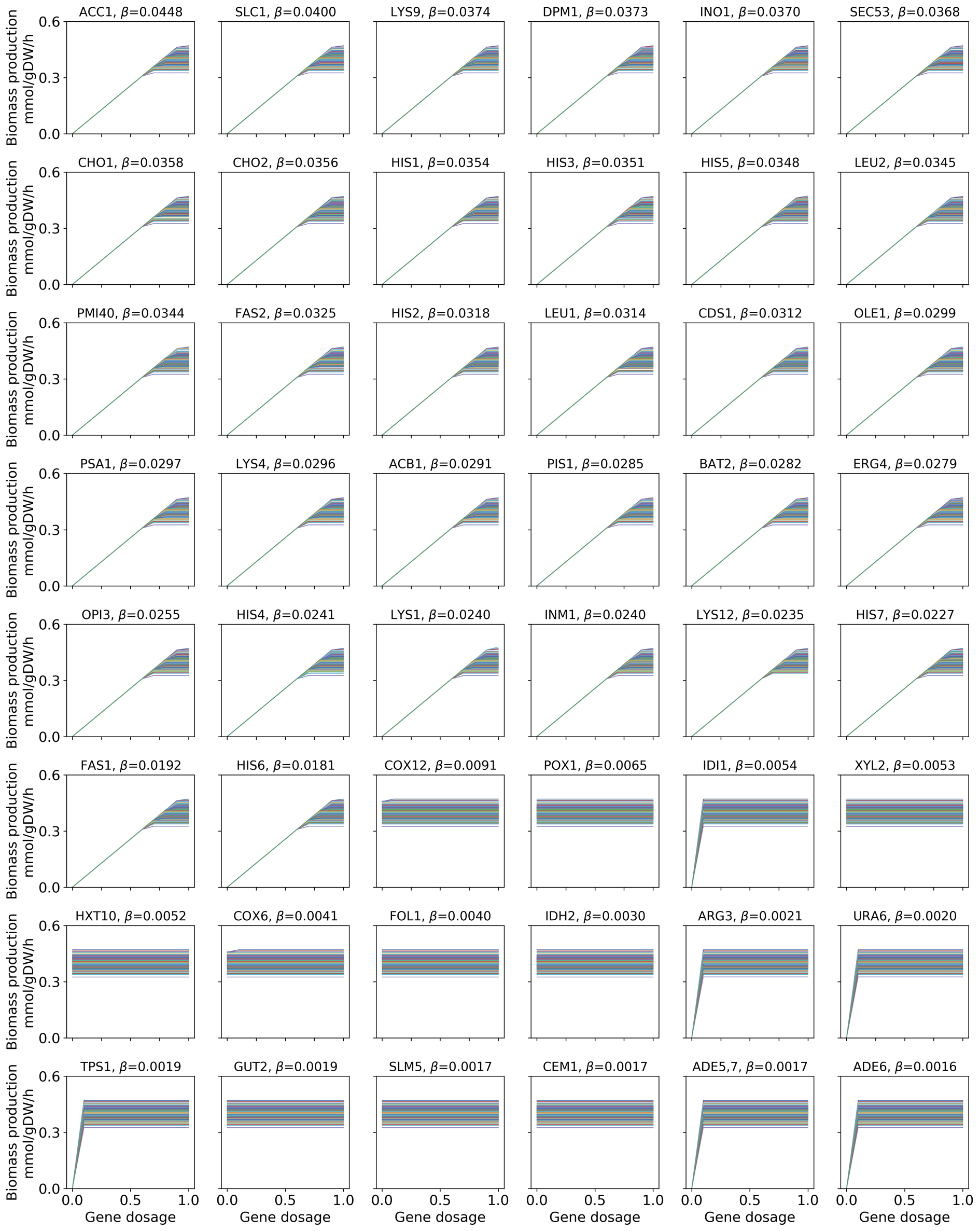

Supplement: S7 Fig — We computed the dosage-response profiles for all predictor genes in 200 genetic backgrounds by individually tuning the corresponding dosage from g = 0 to g = 1 and computing the growth rate with FBA. Observe that i) all top predictors (with β > 0.01) are essential. That is, growth is null if g = 0; ii) that only top predictors display a recurrent dosage-response profile and that iii) the profiles of genes with β < 0.01 are constant in the range mostly accessed by the population 0.7 < g < 1. (TIF) [file pcbi.1011631.s008.tif]

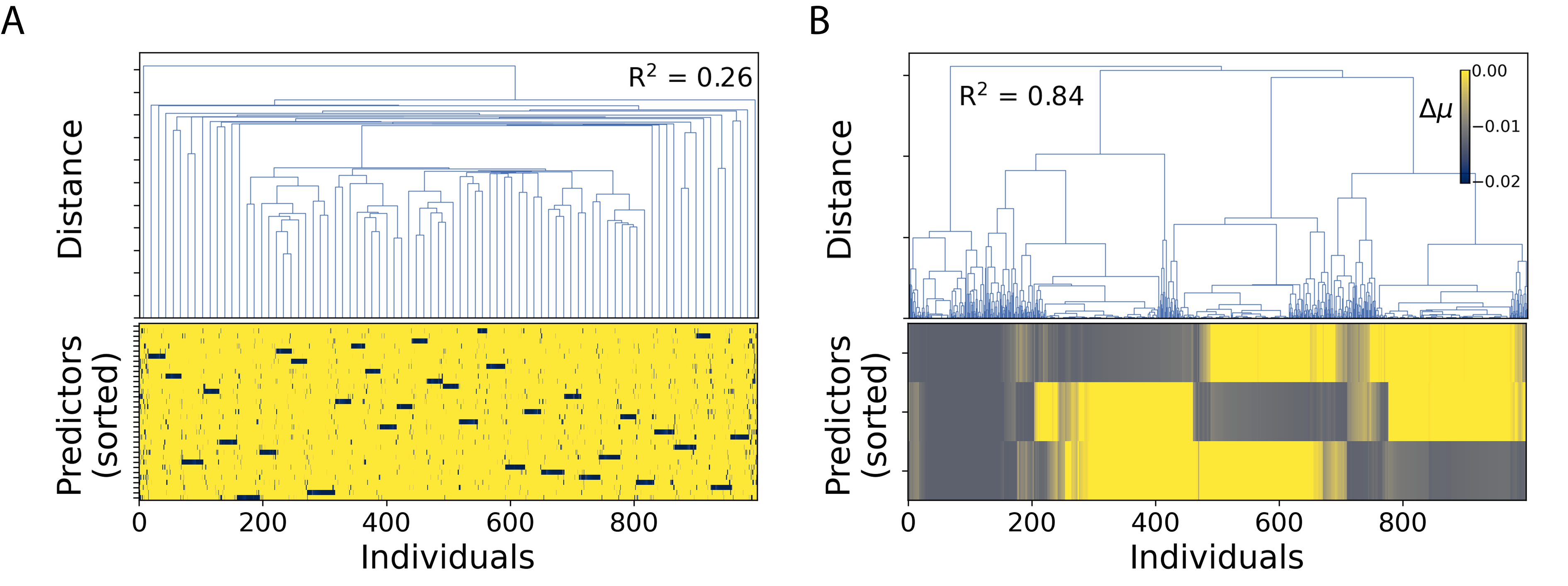

Supplement: S8 Fig — Growth costs (colorbar) caused by individual virtual mutations of enzymes (rows) for 103 different individuals (columns) identify the structure of limiting reactions. The figure displays two populations for which the PGS performance differs (R2 = 0.26 and R2 = 0.84 in A and B, respectively). By clustering individual patterns, we recognize that a more straightforward structure in the dendrogram leads to better prediction (panel B with larger R2 than in panel A with smaller R2). (TIF) [file pcbi.1011631.s009.tif]
