# Supplementary material for: The limitations of phenotype prediction in metabolism
Source: PLoS Comput Biol. 2023 Nov 10;19(11):e1011631. doi: 10.1371/journal.pcbi.1011631 (PMC10664875; doi:10.1371/journal.pcbi.1011631)
Supplement: Attachment — Submitted filename: Response_to_Reviewers_Yuberoetal.pdf [file pcbi.1011631.s011.pdf]

Reviewer #1:

In this manuscript, the Authors use a genome-scale metabolic model of yeast in order to establish a polygenic scoring metric. The Authors apply randomization to generate their datasets, and claim to have used their polygenic score to identify genes, which influence the read-out (predicted specific growth rate) in a dose-dependent manner. I believe the study suffers from fundamental flaws which all should be addressed preceding publication in any scientific journal.

The primary objective of our study is not “to establish a polygenic scoring metric to identify genes, which influence the read-out” rather it is to provide a mechanistic interpretation of the predictions derived from a polygenic risk score (PGS) and demonstrate how the PGS for growth rate prediction is articulated in metabolic models. This emphasis is consistently reiterated in several parts of the manuscript:

> Initial submission lines 64-68*: Our analysis clarifies why individual genes act as predictors in this score and how intrinsic characteristics, that is, of the GP metabolic map, such as pleiotropy or epistasis, and extrinsic, that is, aspects of the population and the environment, are combined to determine the strength of predictability.*

> Initial submission lines 92-94: *Thus, the whole procedure produces a dataset of both genetic and phenotypic variations in the context of a metabolic model, which we can dissect to explain how the system works as a whole.*

> Initial submission lines 240-242*: This feature and the fact that the genetic variation produced is not restricted as in the case of natural populations allows us to study in depth how and why a PGS can predict the phenotype…*

Major comments

Many of the points listed below will point into the main issue of the study as I see it: application of statistical methods without addressing biological considerations.

To remove bias, linked to specific growth environment(s), Authors use some randomization-based pipeline to generate, as they call them, environments “representing evolutionary history” of yeast. Judging by the description in the Methods, these environments are essentially a panel of supplements to the very same medium.

I do not treat this as a valid representation of the potential environments of S. cerevisiae – there are plenty of carbon and nitrogen sources these cells can consume, and the Authors only use glucose as a primary and very abundant (20 mmol/gDW/h, around the maximal uptake rate S. cerevisiae ever show) carbon source. What about the dozen(s) of other carbon sources S. cerevisiae can (co)consume?

We would like here to highlight to the reviewer the distinction between standard medium (incorporating glucose) and minimal medium (a baseline for generating many different media as noted below). This is clearly described in the Methods section ('Growth media and environmental variability' section, lines 389-406) of our manuscript. Thus, “we don't just use glucose as a primary carbon source”, as the referee argues. Second, to mimic natural yeast environments to obtain reference fluxes, we included ALL potential carbon AND nitrogen sources in various combinations, precisely 20,000 combinations (lines 346-347). This is an already validated protocol to mimic natural environments (for example, see reference Wang and Zhang 2009, lines 583-584). Third, the population of study is growing in a fixed standard medium:

> Initial submission lines 85-88: *we simulated growth under a number of environmental and genetic conditions that resembles a likely evolutionary history of yeast metabolism (Methods and Fig. S1). The maximum flux exhibited by each reaction across all of those conditions designates a reference flux for the genetic variation.*

> Initial submission lines 98-100: *By fixing the growing medium in standard nutrients, we controlled for environmental effects on the phenotype.*

Initial submission line 107-109: *That most of the fluxes, in the standard medium considered, are inactivated explains this large number of genes with no effect.*

Initial submission line 128-129: *Thus, the functional mode operating in the standard medium effectively selects a domain within the entire architecture of growth.*

To clarify this, we have slightly modified initial submission line 88 and the Methods section on "Growth media and environmental variability" lines 414 to 420 ("tracked changes manuscript" lines from now on).

Moreover, the Authors try to avoid generating "rich" media, which by itself breaks down their whole idea of comprehensive representation of different natural environments.

Our intention is not to avoid the use of "rich media" in general, but rather to address the issue of "unrealistically rich media" as in metabolic models one can generate arbitrarily large growth rates. In fact, we simply considered an upper limit given by the currently used standard medium with plenty of oxygen and glucose in the unconstrained model. This medium is widely adopted in current research as a baseline for comparison and benchmarking purposes.

Initial submission lines 400 to 403: *To avoid arbitrarily including rich media, we consider those with richness less than or equal to that of the standard media. In addition, we discarded media that support biomass production rates <70% of those of the standard medium to avoid possible natural or model artifacts related to our implementation of quantitative mutations…*

To clarify this, we have slightly extended the methods section in lines 414-416 of the new version.

More to the point of defining the growth environments. Inorganic salts are allowed to take up ad libitum, glucose – pretty much as well, but why oxygen uptake is the only hard flux constraint here? Intuitively, this "locks" the phenotype space to only include fermentative phenotypes, which might be not at all reasonable when, e.g. tested mutants exhibit low specific growth rate. Moreover, the numbers for uptake bounds themselves are not explained (no references or other support for the values of 2 and 20 mmol/gDW/h for oxygen and glucose, respectively)

Note again the distinction between the standard and the minimal media and the explicit reference for a customary environment manipulation in yeast metabolic models Wang and Zhang (2009) on line 393. Moreover, we indeed restrict the phenotype space to respiratory phenotypes by considering a realistic oxygen import rate. By doing so, we aim to focus on physiologically relevant metabolic behaviors that align with respiratory functions. With respect to the constraints on inorganic salts, their <u>import is not unconstrained</u> in our experimental setup. In each random medium, only a limited number of inorganic salts are selected for import. Import of inorganic salts occurs only in those specific cases, and even then, the mean import rate is explicitly defined as 10mmol/gDW/h (the mean value of a uniform distribution), as stated in lines 392-397 of our manuscript. Finally, the values of 20 mmol/gDW/h are commonly used in the FBA literature and correspond to rich nutrient-rich conditions, e.g., YPD. See reference Duarte NC (line 524), Poyatos JF (line 594), and, for instance, Harrison, R., Papp, B., Pal, C., Oliver, S. G. and Delnert, D. (2007) Plasticity of genetic interactions in metabolic networks of yeast. *PNAS* **104**, 2307—2312 (Table S8) for explicit details.

Overall, we have extended the methods section in lines 414-420 of the new version to clarify this.

Eventually, the Authors construct a polygenic score for predicting growth rates, and it does not perform so well ($R2 < 0.3$). My first feeling why the R2 score is low is the following: based on the description of the routines, is that the Authors do not address the issue stemming from the presence of isozymes – in the metabolic model, preventing the flux that uses a (biologically known) major isozyme will have little-to-no effect if there exist alternative isozymes and/or cofactors that the enzymes use. Authors briefly touch upon this in the Discussion (Lines 254-255) but there are no clear signals this is resolved in the current study.

The validity of these models relies on precisely including gene reaction rules to accurately model the presence of isozymes and coenzymes. We have mentioned and discussed these rules as early as the first results section of our manuscript. It is unfortunate that the reviewer did not positively acknowledge our quantitative interpretation of these rules, which serves as valuable support for our approach. We believe that highlighting the significance of these rules from the beginning of the manuscript is essential for readers to understand the robustness and reliability of our methodology.

> Initial submission lines 81-82: … *Gene Reaction Rules (GRR): Boolean relationships between enzymes that define which and how they participate in the reactions (Fig. 1B, Methods and Fig. S1).*

Moreover, understanding the precise value of R2 is the MAIN objective of this manuscript, and we have reiterated this point extensively throughout the paper.

> Initial submission lines 64-68*: Our analysis clarifies why individual genes act as predictors in this score and how intrinsic characteristics […] and extrinsic […] are combined to determine the strength of predictability.*

> Initial submission lines 92-94*: Thus, the whole procedure produces a dataset of both genetic and phenotypic variations in the context of a metabolic model, which we can dissect to explain how the system works as a whole.*

> Initial submission lines 240-242*: This feature and the fact that the genetic variation produced is not restricted as in the case of natural populations allows us to study in depth how and why a PGS can predict the phenotype…*

We have clarified all this in the results section lines 82-84 and 109-111 of the new version of the manuscript.

The major insight of the manuscript (Section "Few metabolic functions limit growth") is, in my opinion, trivial.

We explicitly mention and reference the likelihood of several metabolic pathways simultaneously constraining growth (apparently trivial for the reviewer), in the results section 3:

> Initial submission lines 114-115: *One might think that the predictors are distributed among all metabolic activities…*

And we already comment on the "expected" link between genetic predictors and biomass precursors

> Initial submission lines 118-119: *This group of metabolites feeds the biomass reaction, which defines the architecture of the trait --in this case growth-- in metabolic reconstructions.*

> Initial submission lines 261-264: *Note, however, that these predictors should be necessarily associated with biomass precursors, either directly or in upstream reactions, since the biomass reaction represents the genetic architecture of the trait of interest, growth (Fig. 3AB).*

Line 117-118: "However, we only found a few predictor-enriched metabolic subsystems (Methods, Fig. S3 and Table S1). These subsystems specifically involve the production of 118 biomass precursors.". Naturally this conclusion arises when considering that ALL of these precursors must be made de novo or imported in order to produce biomass, which is the ONLY read-out that Authors use. The point that Authors consider minimal media only does not help here either – a single knockout in a single pathway of making one of the biomass precursors could prevent biomass formation in minimal but not rich media.

Although all genetic predictors are related to biomass precursors (see previous comment), we point out that not all precursors are equally represented, a finding that we consider to be non-trivial:

> Initial submission lines 125-131: *The strongest predictors only contribute significantly to a subset of precursors (11 out of 43), and in some cases, e.g., valine, lysine, etc., this represents the full production that is required for growth. Thus, the functional mode operating in the standard medium effectively selects a domain within the entire architecture of growth.*

While we acknowledge the reviewer's point that all biomass precursors need to be made de novo (since the growing medium is the standard one, not the minimal medium) our result cannot be linked to the case (s)he proposes. First, it is worth noting that our study focuses on gene knock-downs rather than mutant deletions, and none of the metabolic functions are completely blocked:

> Initial submission lines 78-80: *The novelty here is that we can design genetic variation by generating a population of metabolisms in which each member exhibits different alleles determining contrasting gene (expression) dosages.*

Initial submission lines 374-376: *We generate genetic variation by sampling gene (expression) dosages from a probability distribution. Unless otherwise stated, we use a normal distribution with unit mean and standard deviation σ = 0.1.*

Second, our work focuses on yeast growth prediction in the standard medium, and if the conditions change as suggested by the reviewer, genetic predictors might do as well as we further analyzed in the last results section on PGS transportability across media:

Initial submission lines 209-211*: In our final study, we asked to what extent specific growing conditions influence predictive power. These conditions could alter the functional mode of the metabolisms, consequently modifying the PGS.*

Initial submission lines 244-248: *Which genes act as predictors result from the combination of two factors: the quantitative flux required in the reaction(s) associated with the predictor gene and the flux constraints derived from the corresponding genetic variation (Fig. 7A illustrates this combination). The former contributes to the functional mode of metabolism, which incorporates environmental information (growing conditions).*

Minor. Note that there are only 43 predictors (initial submission line 126), we suspect that the referee misread the line number, initial submission line 118, with the number of predictors.

Minor comments

I find the Author's interpretation of Gene-Protein-Reaction (GPR) associations strange (Lines 80-82): "Subsequently, the dosages are quantitatively interpreted in the model by Gene Reaction Rules (GRR): Boolean relationships between enzymes that define which (and how) they participate in the reactions (Fig. 1B, Methods and Fig. S1)." I wonder how Boolean descriptions are supposed to be quantitative, in the meantime capable of acquiring only values of "true" and "false". The Authors claim to give quantitative meaning through applying flux constraints, but this has NOTHING to do with the GPR associations themselves!

Our approach for interpreting GPRs in a quantitative manner is detailed in the supplementary figure S1 and the methods section "Quantitative mutations":

Initial submission lines 353-361: *To find how reducing the dose of an enzyme translates into reduced flux through its reactions relative to reference, we quantitatively interpret gene reaction rules (GRRs). This is necessary because some reactions may require several subunits or only one of several isoenzymes. GRRs can contain operators AND and OR that act on pairs of genes, we consider them equivalent to min and sum, respectively, that act on relative dosages of genes. This approach is similar to those used in noise propagation (Wang and Zhang 2011), or by the Escher package (King et al. 2015). In all cases, the upper/lower limits are always calculated and set according to the reversibility of the reactions, while the limits of ATP maintenance, biomass production, and exchange reactions remain unchanged.*

I do not completely follow why the Authors are exploring these artificial variability/mutation profiles without first consulting a profound panel of natural genetical variability in the same organism, S. cerevisiae, in different ecological niches (Peter 2018 Nature).

As we mention throughout the manuscript, our flexibility in designing the genetic variation is an asset that allows us to explore the genotype-phenotype map more comprehensively. By going beyond observed natural variation, we can gain valuable insights into the underlying mechanisms and interactions that govern the genotype-phenotype relationship. This approach enables us to uncover novel associations and better understand the complexity of the system under investigation:

Initial submission lines 77-80: *Our first goal is to emulate this scenario using an in-silico metabolism of Saccharomyces cerevisiae. The novelty here is that we can design genetic variation by generating a*

population of metabolisms in which each member exhibits different alleles determining contrasting gene (expression) dosages.

Initial submission lines 185-188: *We stress again that one advantage of our approach is that we can generate variation beyond what we might observe in a particular natural situation, where allele frequencies might be constrained by natural selection, genetic drift, etc.*

Initial submission lines 238-242: *The computational model acts as an explicit GP map in which the quantitative interpretation of the GRRs incorporates a fundamental structural layer (Kavvas et al. 2020). This feature and the fact that the genetic variation produced is not restricted as in the case of natural populations allows us to study in depth how and why a PGS can predict the phenotype (growth rate*)

Note that the relationship between environmental or ecological factors and the observed genetic variation in yeast populations is beyond the scope of our manuscript.

Moreover, fluxomics studies of multiple mutants and growth in different conditions (E. coli and S. cerevisiae) are available – why not to make good use of that data?

See the previous comment. We reiterate that the primary aim of our study is to investigate the metabolic aspects that underlie the mechanistic relationship between a phenotype and the statistical prediction methods, specifically focusing on deriving a biological reasoning behind the precise value of R2 of a polygenic risk score (PGS). By examining the metabolic models and understanding the biological mechanisms driving growth rate predictions, we aim to provide a comprehensive and mechanistic interpretation of the statistical predictions. This approach allows us to establish a solid biological foundation for the predictive performance of PGS and deepen our understanding of the genotype-phenotype relationship in the context of metabolism

We appreciate the significance of fluxomics research in generating and improving solid metabolic models. While we acknowledge the importance of such research as a foundation for our work, we want to emphasize that the focus of our manuscript is not on conducting fluxomics experiments or directly improving metabolic models. Again, our primary objective is to provide a mechanistic interpretation of the predictions made by PGSs in the context of growth rate prediction. We recognize the broader context in which our study sits, but we aim to contribute specifically to **the understanding of the relationship between PGS, metabolic models, and growth rate prediction**, rather than directly engaging in fluxomics research or model improvement.

Reviewer #2:

Major Comments:

General question – how does this model consider the established feedback mechanisms that exist in yeast to induce biosynthetic machinery under conditions of limited availability of biomass precursors? The most relevant example is histidine. The lack of histidine can be sensed by GCN2, and biosynthetic machinery transcriptionally activated by GCN4. Is there a way in which this type of regulation can be included in the model?

We appreciate the reviewer's comment regarding the limitation of generic metabolic models in capturing genetic regulation. It is true that genetic regulation plays a crucial role in shaping biological phenotypes. However, in our study, we specifically focus on understanding the predictability of growth based on genetic variation within a population of metabolisms. While genetic regulation is an important aspect to consider, we make the assumption that regulatory mechanisms are functioning appropriately and unhindered in our analysis. We consider that all possible and known alternate biosynthetic routes can be eventually required and activated to support growth. By taking this approach, we aim to capture the potential metabolic capabilities of the system under different genetic variations. Although the genetic regulation aspect is not explicitly incorporated in our study, we believe that our approach still provides valuable insights into the predictability of growth based on genetic variation.

We now explicitly mention this limitation and its relationship with our work in the supplementary material section "Flux balance analysis".

How do the authors explain the case of mannan production. The text states that upstream genes in the pathway are predictors, however, the final enzyme responsible for production of the terminal metabolite "have null effect sizes". Does this imply that the production of the terminal metabolite is not causative for the growth effect? If so, do the preceding metabolites have alternative metabolic routes? Could the production/consumption of co-factors be important (NAD+, etc)? The same information is desired for erg4 and sterol production.

Although in the results section #2 "Few metabolic functions limit growth" we described the cases of mannan and sterol production, the reasoning of why some upstream genes instead of the final ones appear as growth predictors is discovered in the following sections and summarized in the discussion:

> Initial submission lines 244-251: *Which genes act as predictors result from the combination of two factors: the quantitative flux required in the reaction(s) associated with the predictor gene and the flux constraints derived from the corresponding genetic variation (Fig. 7A illustrates this combination). The former contributes to the functional mode of metabolism, which incorporates environmental information (growing conditions). The latter integrates information on allele frequencies in the population with the inherent architecture of metabolism and its reference bounds, a product of the environmental and genetic conditions experienced during its evolutionary history.*

In our study, we observe that if upstream genes involved in mannan production are growth-limiting, while the final enzyme (*pmt1-6*) is not, it can be attributed to the reaction flux limits. The upstream genes are operating close to their reference flux constraint, which is determined by historical adaptations. On the other hand, the final enzyme is operating well below its maximum capacity. Therefore, the limited genetic variation present in the population does not significantly restrict mannan production through *pmt1-6*. This observation highlights the importance of considering reaction flux limits and their impact on metabolic pathways and phenotypic outcomes. We emphasize this now in the discussion (lines 270-272; "tracked changes manuscript" lines from now on).

Can the authors provide additional details on the effect of genetic variation on the growth of the population?

Gradual increases in variation lead to a notable consequence: a specific set of predictors (shown in red in Fig. 5C) begins to exert a stronger limitation on growth. While this enhances prediction accuracy (reflected in an increase in R2), it does not have the same positive effect on growth, as the limiting fluxes become more constricted. Additionally, beyond the maximum point, a new set of predictors emerges (indicated in green in Fig 5C), which complicates the prediction capability (involving more factors that influence R2) and further reduces growth. We have extended a bit the explanation of this in lines 201-205.

The authors should include some metrics for predictor genes across experimental conditions – for example his4 appears to have been identified several times. "We also observe that other genes appear recurrently as strong predictors in specific, generally poor environments (Fig. 6B), and whose appearance leads to particularly strong PGS performance with up to R2 = 0.56 (Fig. 6C)." It might be useful to know what these genes are, or explicitly mention that those are the genes shown in Fig. 6D.

The primary focus of our manuscript is to analyze the reasons behind and the extent to which a polygenic risk score (PGS) can predict the growth rate of a mutant metabolism under specific conditions, namely a history of adaptations and a fixed standard medium. While we acknowledge the potential importance and utility of the specific genes we identified as growth-limiting for other researchers, our study primarily aims to investigate the mechanistic interpretation of PGS and its ability to anticipate growth rates. We believe that our findings contribute valuable insights into the relationship between genetic variations, metabolic models, and growth predictions, providing a foundation for further research in this area.

As pointed out by the reviewer, we now explicitly mention that all predictor genes observed (in the standard and random media) are listed in Fig. 6D; line 232 in the new version.

The authors utilize a LASSO regression for generation of PGS and identification of genes with a large beta. In the methods, the authors state "That effect sizes show a bimodal distribution makes our results robust to the application of other regularization, or feature selection methods (Fig. 2E)". It would be interesting to test if other regression models (perhaps ridge or elastic net) yield similar number of genes with large beta.

Lasso, Ridge and ENet (combining lasso and ridge) are all Bayesian interpretations of a linear regression with different priors. Although the size effects and the R2 might quantitatively depend on the type of regression, the qualitative remains constant, and thus the major claims of this manuscript. We follow the referee's advice and tested these other models. We now explicitly mention the R^2 values using these approaches in the methods section "Polygenic Score" (lines 459-463)

Minor comments:

Some mention should be made about how the model handles multi-gene protein complexes which require each protein to function. Does altering gene dosage of a catalytic subunit reflect a growth/biomass defect for other components of the complex?

Indeed, we do propagate defects on gene dosages to the functioning of coenzymes, this is precisely the quantitative interpretation of gene reaction rules which are mentioned as early as the first results section:

> Initial submission lines 81-82: … *Gene Reaction Rules (GRR): Boolean relationships between enzymes that define which and how they participate in the reactions (Fig. 1B, Methods and Fig. S1).*

In response to comments from reviewers #1 and #2, we have provided further clarification in the result section (lines 82-84) in the revised version of the manuscript.

Line 129 – "especial" to "special"

Corrected.

Reviewer #3:

What are the main claims of the paper and how significant are they for the discipline?

- The authors attempted to study the mechanism behind the GWAS scores. To this end, they have chosen to use a genome-scale metabolic reconstruction of the

- Are these claims novel? If not, which published articles weaken the claims of originality of this one?
  - Yes
- Are the claims properly placed in the context of the previous literature? Have the authors treated the literature fairly?
  - Not fully. Authors did position their work within the GWAS literature; however, they have poorly addressed the literature in terms of their biological findings. What is the accuracy of the GWAS predictions based on the literature? Have the genes identified in the analysis been shown to modulate the growth? Are there genes that have not been identified as essential for growth but are known in the literature to be?
  - Furthermore, there is unclarity on how the fluxes were calculated and authors do not comment on weather the flux vectors used were unique solutions. For genome-scale models it is known that the standard FBA or even pFBA does not guarantee a unique solution for all reactions in the network. It does uniquely solve the objective function (for example growth), but all the other fluxes, especially internal ones, are subject to uncertainty usually studied using FVA or random flux sampling methods. Furthermore, the use of pFBA is not discussed in the paper, rather can be discovered in the code

itself. pFBA is a method that can significantly improve the results of simulations but only for organisms that do optimize growth above all (see Lewis, 2010, https://doi.org/10.1038/msb.2010.47).

    - The resulting GWAS model has very low predictability score, even in the test data. What is the benefit for its application? Can it be applied? What reservations should be considered? The description is very superficial in this aspect.

- Do the data and analyses fully support the claims? If not, what other evidence is required?
    - Following on the previous statement, further analysis needs to be done on the accuracy of the predictions (albeit the accuracy might be lowered by a low quality of the genome-scale reconstruction). Nonetheless, if the method is to be used by a wider community, these questions need to be addressed.
    - Authors make several claims based on the flux vector analysis. However, if the flux vector is non-unique the analysis can be very superficial.
- Would additional work improve the paper? How much better would the paper be if this work were performed and how difficult would it be to do this work?
    - Text should be clarified in regard to flux vector uniqueness and the methods used to ensure it. Furthermore, authors could spend some more time to compare their findings with the biology literature to attest the accuracy of their method. Both should not take a lot of time or resources to complete.
    - However, if the flux vector is non-unique authors would be encouraged to explore methods that can ensure the uniqueness and re-do their analysis to draw the correct conclusions.
- *PLOS Computational Biology* encourages authors to publish detailed protocols and algorithms as supporting information online. Do any particular methods used in the manuscript warrant such treatment? If a protocol is already provided, for example for a randomized controlled trial, are there any important deviations from it? If so, have the authors explained adequately why the deviations occurred?
    - The supporting data is very transparent. And allows one to study the code and methods in depth.
- Are original data deposited in appropriate repositories and accession/version numbers provided for genes, proteins, mutants, diseases, etc.?
    - Yes
- Has the author-generated code that underpins the findings been made publicly available?
    - Yes
- Are details of the methodology sufficient to allow the experiments to be reproduced?
    - Yes, as the code is given and can be re-run. The results can be recalculated from it. However, based purely on the methods description in the paper, one would not be able to reproduce the algorithm/code behind it as several details in the descriptions are lacking.
- Is the manuscript well organized and written clearly enough to be accessible to non-specialists?
    - Manuscript could benefit from additional language checks and simplifications for non-specialists to appreciate the work. Some sentences are difficult to follow.
- Does the paper use standardized scientific nomenclature and abbreviations? If not, are these explained at the first usage?
    - Yes

The primary objective of our study is to investigate the metabolic aspects that underlie the mechanistic relationship between a phenotype and statistical prediction methods, with a specific focus on understanding the biological reasoning behind the precise value of R2 for a polygenic risk score (PGS). Our research involves a thorough examination of metabolic models to gain insights into the biological mechanisms driving growth rate predictions. By doing so, we aim to provide a comprehensive and mechanistic interpretation of the statistical predictions, thus establishing a robust biological foundation for the predictive performance of a PGS. In sum, this study aims to deepen our understanding of the genotype-phenotype relationship in the context of metabolism.

It is essential to note that our approach does not specifically address existing literature concerning specific biological findings, nor do we inquire about the direct applicability of our findings in a particular scenario. Instead, our emphasis lies on contributing valuable insights into the underlying biological mechanisms supporting the statistical predictions. While we acknowledge the potential significance of some specific predictions in our research, we want to clarify that the primary focus of our manuscript is not on testing our PGS predictions.

Furthermore, the main concerns raised by reviewer #3 pertain to the uniqueness of solutions obtained from flux balance analysis (FBA) and the utilization of parsimonious FBA (pFBA). We would like to clarify that our approach ensures **coherent solutions within a population**, similar to the concept of Minimization of Metabolic Adjustment (MOMA): we make sure that FBA does not find solutions that are too distinct between members of the same population growing in the same medium as shown in Fig S2B (that describes the variation in solution fluxes, see caption). We clarify this issue in lines 102-104, main text. Furthermore, we have now explicitly mentioned and referenced the use of pFBA in the methods section to provide more methodological details (lines 344-345). We have made an effort to ensure the reproducibility of this work by providing the necessary code. We also edited several parts of the manuscript (highlighted in magenta).

Regarding the limited predictive power of the specific polygenic risk score (PGS) discussed, we emphasize that the main focus of our manuscript is to understand the interpretability of PGS in the context of metabolic models, rather than maximizing its predictive ability. Therefore, the general claims and conclusions of our study do not rely on the specificities of the model, including the metabolic network, the flux solution search algorithm, the R2 value, or the true growth prediction ability of certain genes in specific experimental conditions. We hope this clarification addresses the concerns raised by the reviewer and highlights the broader focus and implications of our research.
