# Supplementary material for: The limitations of phenotype prediction in metabolism
Source: PLoS Comput Biol. 2023 Nov 10;19(11):e1011631. doi: 10.1371/journal.pcbi.1011631 (PMC10664875; doi:10.1371/journal.pcbi.1011631)
Supplement: Attachment — Submitted filename: review.docx [file pcbi.1011631.s010.docx]

- What are the main claims of the paper and how significant are they for the discipline?
  - The authors attempted to study the mechanism behind the GWAS scores. To this end, they have chosen to use a genome-scale metabolic reconstruction of the
- Are these claims novel? If not, which published articles weaken the claims of originality of this one?
  - Yes
- Are the claims properly placed in the context of the previous literature? Have the authors treated the literature fairly?
  - Not fully. Authors did position their work within the GWAS literature; however, they have poorly addressed the literature in terms of their biological findings. What is the accuracy of the GWAS predictions based on the literature? Have the genes identified in the analysis been shown to modulate the growth? Are there genes that have not been identified as essential for growth but are known in the literature to be?
  - Furthermore, there is unclarity on how the fluxes were calculated and authors do not comment on weather the flux vectors used were unique solutions. For genome-scale models it is known that the standard FBA or even pFBA does not guarantee a unique solution for all reactions in the network. It does uniquely solve the objective function (for example growth), but all the other fluxes, especially internal ones, are subject to uncertainty usually studied using FVA or random flux sampling methods. Furthermore, the use of pFBA is not discussed in the paper, rather can be discovered in the code itself. pFBA is a method that can significantly improve the results of simulations but only for organisms that do optimize growth above all (see Lewis, 2010, <https://doi.org/10.1038/msb.2010.47>).
  - The resulting GWAS model has very low predictability score, even in the test data. What is the benefit for its application? Can it be applied? What reservations should be considered? The description is very superficial in this aspect.
- Do the data and analyses fully support the claims? If not, what other evidence is required?
  - Following on the previous statement, further analysis needs to be done on the accuracy of the predictions (albeit the accuracy might be lowered by a low quality of the genome-scale reconstruction). Nonetheless, if the method is to be used by a wider community, these questions need to be addressed.
  - Authors make several claims based on the flux vector analysis. However, if the flux vector is non-unique the analysis can be very superficial.
- Would additional work improve the paper? How much better would the paper be if this work were performed and how difficult would it be to do this work?
  - Text should be clarified in regard to flux vector uniqueness and the methods used to ensure it. Furthermore, authors could spend some more time to compare their findings with the biology literature to attest the accuracy of their method. Both should not take a lot of time or resources to complete.
  - However, if the flux vector is non-unique authors would be encouraged to explore methods that can ensure the uniqueness and re-do their analysis to draw the correct conclusions.
- *PLOS Computational Biology* encourages authors to publish detailed protocols and algorithms as supporting information online. Do any particular methods used in the manuscript warrant such treatment? If a protocol is already provided, for example for a randomized controlled trial, are there any important deviations from it? If so, have the authors explained adequately why the deviations occurred?
  - The supporting data is very transparent. And allows one to study the code and methods in depth.
- Are original data deposited in appropriate repositories and accession/version numbers provided for genes, proteins, mutants, diseases, etc.?
  - Yes
- Has the author-generated code that underpins the findings been made publicly available?
  - Yes
- Are details of the methodology sufficient to allow the experiments to be reproduced?
  - Yes, as the code is given and can be re-run. The results can be recalculated from it. However, based purely on the methods description in the paper, one would not be able to reproduce the algorithm/code behind it as several details in the descriptions are lacking.
- Is the manuscript well organized and written clearly enough to be accessible to non-specialists?
  - Manuscript could benefit from additional language checks and simplifications for non-specialists to appreciate the work. Some sentences are difficult to follow.
- Does the paper use standardized scientific nomenclature and abbreviations? If not, are these explained at the first usage?
  - Yes
